# Hodgkin Lymphoma Cell Lines and Tissues Express mGluR5: A Potential Link to Ophelia Syndrome and Paraneoplastic Neurological Disease

**DOI:** 10.3390/cells12040606

**Published:** 2023-02-13

**Authors:** Sofia Schnell, Ellen Knierim, Petra Bittigau, Jakob Kreye, Kathrin Hauptmann, Patrick Hundsdoerfer, Susanne Morales-Gonzalez, Markus Schuelke, Marc Nikolaus

**Affiliations:** 1 NeuroCure Cluster of Excellence, Charité-Universitätsmedizin Berlin, 10117 Berlin, Germany; 2 Department of Neuropediatrics, Charité-Universitätsmedizin Berlin, 10117 Berlin, Germany; 3 Center for Chronically Sick Children, Charité-Universitätsmedizin Berlin, 10117 Berlin, Germany; 4 Berlin Institute of Health (BIH), 10178 Berlin, Germany; 5 Department of Neurology and Experimental Neurology, Charité-Universitätsmedizin Berlin, 10117 Berlin, Germany; 6 German Center for Neurodegenerative Diseases (DZNE), 37075 Göttingen, Germany; 7 Institute of Pathology, Charité-Universitätsmedizin Berlin, 10117 Berlin, Germany; 8 Helios Klinikum Berlin-Buch, Kinder-und Jugendmedizin, 13125 Berlin, Germany

**Keywords:** metabotropic glutamate 5 receptor, anti-mGluR5 encephalitis, neuroimmunology, pediatric neurology, pediatric oncology, transcriptome analysis, Hodgkin lymphoma, Ophelia syndrome

## Abstract

Ophelia syndrome is characterized by the coincidence of severe neuropsychiatric symptoms, classical Hodgkin lymphoma, and the presence of antibodies to the metabotropic glutamate 5 receptor (mGluR5). Little is known about the pathogenetic link between these symptoms and the role that anti-mGluR5-antibodies play. We investigated lymphoma tissue from patients with Ophelia syndrome and with isolated classical Hodgkin lymphoma by quantitative immunocytochemistry for mGluR5-expression. Further, we studied the L-1236, L-428, L-540, SUP-HD1, KM-H2, and HDLM-2 classical Hodgkin lymphoma cell lines by FACS and Western blot for mGluR5-expression, and by transcriptome analysis. mGluR5 surface expression differed significantly in terms of receptor density, distribution pattern, and percentage of positive cells. The highest expression levels were found in the L-1236 line. RNA-sequencing revealed more than 800 genes that were higher expressed in the L-1236 line in comparison to the other classical Hodgkin lymphoma cell lines. High mGluR5-expression was associated with upregulation of PI3K/AKT and MAPK pathways and of downstream targets (e.g., *EGR1*) known to be involved in classical Hodgkin lymphoma progression. Finally, mGluR5 expression was increased in the classical Hodgkin lymphoma-tissue of our Ophelia syndrome patient in contrast to five classical Hodgkin lymphoma-patients without autoimmune encephalitis. Given the association of encephalitis and classical Hodgkin lymphoma in Ophelia syndrome, it is possible that mGluR5-expression in classical Hodgkin lymphoma cells not only drives tumor progression but also triggers anti-mGluR5 encephalitis even before classical Hodgkin lymphoma becomes manifest.

## 1. Introduction

Classical Hodgkin lymphoma is a B-cell lymphoma characterized by the presence of a few giant multinucleated Hodgkin and Reed–Sternberg cells [1]. These cells make up less than 1% of the infiltrated lymphoid tissue and are surrounded by inflammatory cells that form the tumor microenvironment [1,2]. Hodgkin and Reed–Sternberg cells express the CD30 surface marker and thereby define classical Hodgkin lymphoma. The disease is divided into the subtypes (i) nodular sclerosis, (ii) mixed cellularity, (iii) lymphocyte-depleted, and (iv) lymphocyte-rich, which represent the majority of Hodgkin lymphoma [2]. Although Hodgkin lymphoma accounts for only 5–6% of all childhood cancers, it is the most frequent neoplasia in adolescents and young adults between 15 and 19 years of age with a second peak in elderly individuals [3,4,5].

Classical Hodgkin lymphoma is not only known to be associated with viral infections and autoimmune diseases [6,7,8], but also with atypical immune-mediated phenomena such as paraneoplastic neurological syndromes [9,10,11]. These are often caused by an antibody-mediated response against so-called “onconeural” antigens, e.g., antigens that are expressed by both the nervous system and tumor [12]. Thus, ectopic expression of these antigens by classical Hodgkin lymphoma may trigger a misdirected autoimmune response against neuronal structures due to molecular mimicry [13,14]. Paraneoplastic neurological symptoms can precede, accompany, or occur in the wake of classical Hodgkin lymphoma [6,15,16]. Examples are the paraneoplastic cerebellar degeneration (PCD) syndrome, subacute cortical cerebellar degeneration (SCCD), and limbic encephalitis (LE) [9,14,17]. A form of the latter was first described as “Ophelia syndrome” by Ian Carr (1982) [14,18,19,20]. It primarily and exceedingly affects children and young adults, who develop severe psychosis with extensive hallucinations, behavioral changes, cognitive dysfunction, seizures, movement disorders, and sleep disturbance [10,18,21,22]. Lancaster et al. (2011) described pathogenic autoantibodies in Ophelia syndrome that target the metabotropic glutamate receptor 5 (mGluR5) and cause a decrease in mGluR5 density on neurons [19]. Early recognition of this anti-mGluR5 encephalitis by cerebrospinal fluid antibody screening is crucial, as patients respond favorably to antibody removal. Most patients recover and benefit from thorough follow-up with early detection of classical Hodgkin lymphoma [18,19]. Although lymphoma occurs in half of all Ophelia cases, there are no reports that have investigated the pathophysiological or functional association between anti-mGluR5 encephalitis and classical Hodgkin lymphoma [18]. Therefore, we set out (i) to investigate the expression of mGluR5 in patients with classical Hodgkin lymphoma (some with and some without a prior encephalitis), (ii) to investigate mGluR5 positivity on CD30^+^ Hodgkin and Reed–Sternberg cells from biopsy material of these patients, and (iii) to analyze the transcriptome of various established classical Hodgkin lymphoma cell lines for a correlation between mGluR5 mRNA expression and downstream target activation that might promote tumor growth.

## 2. Materials and Methods

### 2.1. Patient

**Figure 1:**
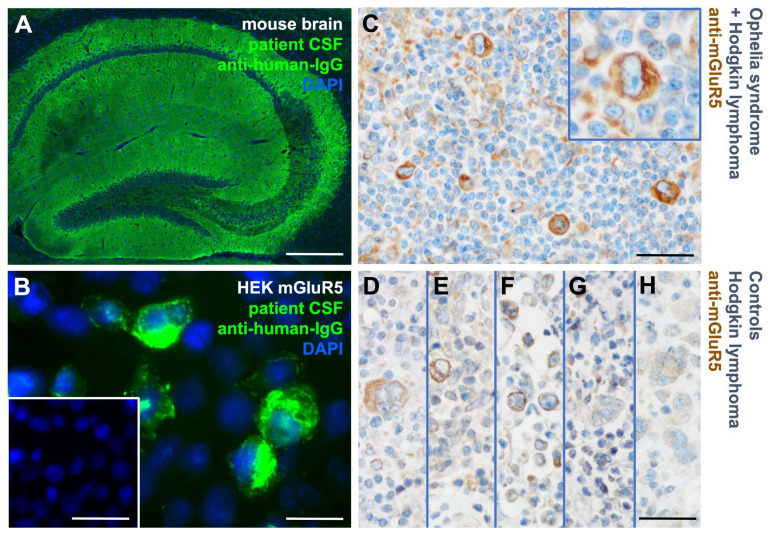
Detection of anti-mGluR5 antibodies in the cerebrospinal fluid and of mGluR5 protein expression in the tumor cells of a patient with Ophelia syndrome. (**A**) Immunostaining of fresh frozen mouse hippocampus with the patient’s cerebrospinal fluid depicts reactivity typical for anti-mGluR5 antibodies. (**B**) Patient’s cerebrospinal fluid immunostaining on mGluR5-expressing HEK293T cells shows clear reactivity in contrast to control (inset). (**C**) ICC with commercial anti-mGluR5 antibodies on a tumor biopsy specimen of the patient after developing classical Hodgkin lymphoma reveals mGluR5^+^ Hodgkin and Reed–Sternberg cells. The inset depicts a magnified multinuclear Reed–Sternberg cell. (**D**–**H**) Biopsy specimens from patients with classical Hodgkin lymphoma but without encephalitis (controls) demonstrate only weak or absent anti-mGluR5 reactivity. Size bars: 100 µm (**A**, inset of **B**, **C**–**H**), 20 µm (**B** and inset of **C**).

We diagnosed anti-mGluR5 encephalitis (Ophelia syndrome) in a 15-year-old boy who presented with acute psychosis, severe encephalopathy, and autonomic dysregulation. Antibody screening revealed the presence of anti-mGluR5 IgG-antibodies in cerebrospinal fluid and serum (Figure 1A,B). Full recovery was only achieved after 22 weeks of ICU treatment and immunotherapy including methylprednisolone, IV immunoglobulins, immunoadsorption, IV and intrathecal rituximab as well as IV bortezomib application. At the time of Ophelia syndrome manifestation, we extensively investigated our patient for the presence of Hodgkin lymphoma including serial abdominal ultrasounds, chest X-ray, whole body MRI, and PET-MRI, as well as laboratory investigations (βHCG, NSE, LDH, uric acid), but did not detect any abnormalities. However, sixteen months later, the patient developed advanced classical Hodgkin lymphoma of the nodular sclerosing subtype, which responded to EuroNet-PHL protocol treatment [23], obtaining complete remission.

### 2.2. Immunological Studies on Tumor Tissue from Patients and Controls

We examined samples obtained from the lymph nodes of the index patient and tissue from n = 5 age-matched classical Hodgkin lymphoma-cases without autoimmune encephalitis. Staining was performed on 3-μm tissue sections from formalin-fixed, paraffin-embedded (FFPE) lymph node biopsy specimens using an automated slide staining system (BenchMark XT, Ventana Medical Systems, Tucson, AZ, USA). We performed heat-induced epitope retrieval (Cell Conditioning 1, Ventana), primary staining with anti-mGluR5 (1:250, Cat# PA5-33823, ThermoFisher, Waltham, MA, USA, or Cat# ab27190, Abcam, Cambridge, UK) and automated secondary antibody staining with the DAB detection kit (iVIEW, Ventana Medical Systems).

### 2.3. Cell Lines and Culture Conditions

We used standard cell lines from classical Hodgkin lymphoma patients of the nodular sclerosing (L-428, HDLM-2, L-540, SUP-HD1) or mixed cellularity (KM-H2, L-1236) subtypes, established from pleural effusions (HDLM-2, KM-H2, L-428, SUP-HD1), bone marrow (L-540), or peripheral blood (L-1236) [24,25,26,27,28,29]. Jurkat cells served as negative control. The cells were cultured in RPMI-1640 GlutaMAX (Invitrogen, Waltham, MA, USA) supplemented with 10% fetal bovine serum and 1% penicillin/streptomycin and maintained in a 5% CO_2_ humidified atmosphere at 37 °C. The source of the classical Hodgkin lymphoma cell lines and the links to the accompanying data sheets are provided in the Supplementary Material section.

### 2.4. Immunocytochemistry—Surface Antigen Labeling and Quantification

**Figure 2:**
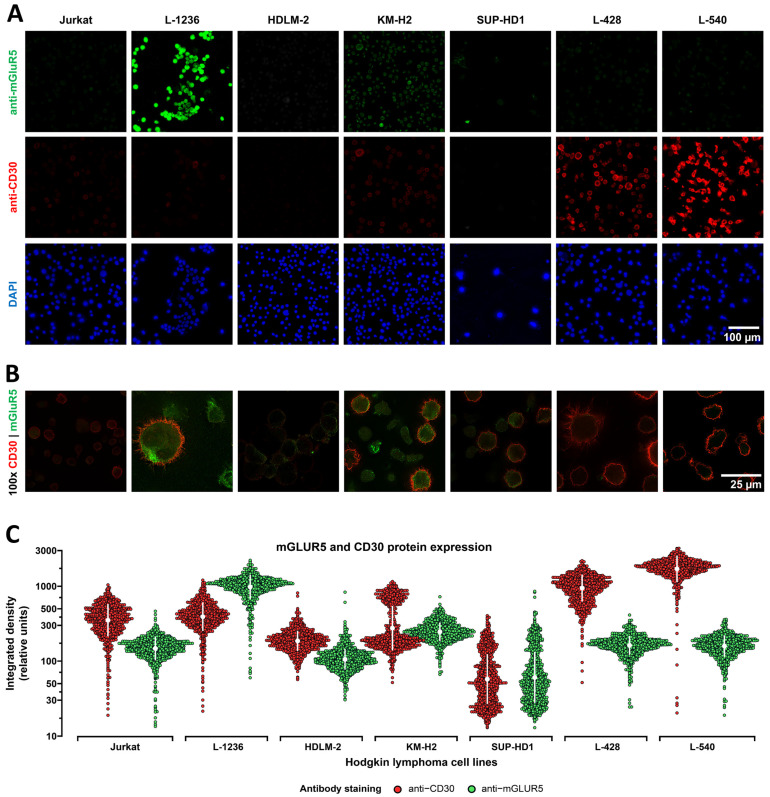
Comparative analysis and quantification of mGluR5 immunostaining in classical Hodgkin lymphoma cell lines. (**A**) Immunostaining of classical Hodgkin lymphoma cell lines using exactly the same imaging parameters to be able to distinguish between the various expression levels for CD30 (red) and mGluR5 (green). Nuclei were stained by DAPI (blue). Jurkat cells, as negative controls, expressed very low levels of CD30 and mGluR5. (**B**) Co-immunostaining of anti-CD30 (red) and anti-mGluR5 (green). (**C**) Dotplots generated with the ggbeeswarm::geom_beeswarm() feature of R!. Staining intensities were given as relative integrated density units of the 8 bit b/w images. A total of 1000 cells from each sample were plotted; the white dot depicts the mean and the white whiskers the SD. The *Y*-axis is adjusted to a log10 scale.

We performed immunocytochemistry (ICC) with patient cerebrospinal fluid on fresh-frozen murine brain sections and on mGluR5-expressing HEK293T cells as described previously [30,31]. Further, we seeded 5 × 10^5^ classical Hodgkin lymphoma cells on poly-L-lysine (Sigma-Aldrich, St. Louis, MO, USA) coated coverslips in 12-well plates. The cells were fixed with 4% paraformaldehyde, blocked with 5% normal goat serum and 2% bovine serum albumin for 1 h at RT and incubated with the primary antibodies against mGluR5 (1:250, Cat# ab76316, Abcam, Cambridge, UK) and CD30 (1:80, Ber-H2, Cat# M0751, Dako, Jena, Germany) O/N at 4°C. Secondary staining was performed with a fluorophore-conjugated anti-rabbit-IgG antibody (Alexa Fluor 488, 1:1000, Cat# A-11008, Invitrogen, Waltham, MA, USA) and anti-mouse antibody (Alexa Fluor 568, 1:250, Cat# A-11004, Invitrogen) for 1 h followed by incubation with 4′,6-diamidino-2-phenylindole (DAPI; 1:1000; Cat# D1306, Invitrogen). Fluorescence was recorded using a THUNDER Imager DMi8 with a Leica DFC9000 GT camera and the LAS(X) software (Leica Microsystems, Wetzlar, Germany). The imaging parameters (illumination light intensity, aperture, exposure time, and camera sensitivity) were kept strictly constant for all recordings. For comparison of anti-mGluR5 and anti-CD30 staining intensities on the classical Hodgkin lymphoma cell lines, we generated four visual fields with a 20× microscope lens on the blue channel (DAPI), the green channel (mGluR5), and the red channel (CD30). We analyzed the layered images using the Fiji/ImageJ v.2.3.0/1.53 software. We used the ROI of the nuclear DAPI signal to find the single cells and radially extended the respective ROIs by 1 μm to also cover the cytosol and cell membrane. We then recorded the integrated densities over all ROIs for each fluorophore from a total of 1000 cells per sample. For this analysis, we started with the cells on the top left and worked our way down the image until we had gathered 1000 cells. The absolute number of the analyzed cells had to be kept constant for statistical reasons. The distributions of the integrated density values were visualized on a dotplot using the R version 4.2.2 from within the RStudio v.1.4.1106 software (Figure 2C).

### 2.5. Western Blot

We extracted protein with cell lysis RIPA Buffer (50 mM Tris-HCl pH 8.0, 150 mM NaCl, 1% NP-40, 0.5% sodium deoxycholate, 0.1% SDS, Complete^®^ protease inhibitors). Samples were treated with 4× LDS loading buffer (NuPAGE^®^) and DTT (NuPAGE^®^ sample reducing agent). A 150-µg sample of each classical Hodgkin lymphoma cell line and Jurkat cells (negative control) were loaded onto a gradient 4–12% NuPAGE^®^ Bis-Tris gel (MES Running buffer; 150 V; XCell Surelock Mini-cell) without boiling. The blots were incubated with primary anti-mGluR5 (monoclonal rabbit antibody, 1:1000, Cat# ab76316, Abcam) and anti-α-Tubulin (1:1000, Cat# MCA77G, Bio Rad, Hercules, CA, USA) antibodies O/N at 4 °C and stained with the corresponding HRP-labeled secondary antibodies: anti-rabbit-IgG (1:2000, Cat# DC03L, Calbiochem, San Diego, CA, USA) and anti-rat-IgG (1:2000, Cat# DC01L, Calbiochem). For detection, we used ECL (Amersham plc, Amersham, UK) and a gel imager (VWR Image Capture Software, Radnor, PA, USA). For verification of the characteristic mGluR5 banding pattern, we loaded protein extracts from the L-1236 Hodgkin lymphoma cell line and 1 µg of mouse brain (positive control) side-by-side and performed a Western blot as described above (Supplementary Figure S1).

### 2.6. Quantitative Reverse-Transcription Quantitative Polymerase-Chain-Reaction

We performed reverse-transcription quantitative polymerase-chain-reactions (RT-qPCR) for relative quantification of *GRM5* gene expression on an ABI 7000 Prism sequence detection system (Applied Biosystems, Waltham, MA, USA) with triplicates in three independent reactions for each sample. Each RT-qPCR reaction contained transcript-specific oligonucleotide primers (for primer sequences see Supplementary Table S1), cDNA of the cell lines of interest, and SYBR Green Master Mix (#4309155; Life Technologies, Carlsbad, CA, USA) in a 20 μL volume. The following cycling conditions were used: 50 °C for 2 min, 95 °C for 10 min followed by 40 cycles of 95 °C for 15 s, 60 °C for 1 min and a final ramp from 60–90 °C for melting curve recording. For generation of a standard curve we prepared a 1:10 serial dilution of 16 steps from the respective PCR-products and ran them under the above conditions. For calculation of the PCR-efficiency we used the n = 7 dilutions that provided the best linear regression line. Relative target gene expression was calculated by the efficiency corrected ΔΔC_t_ method by Pfaffl et al. (2001) using *HPRT* as reference gene [32].

### 2.7. Fluorescence-Activated Cell Sorting (FACS)

For FACS analysis, a standard staining procedure was applied. Each classical Hodgkin lymphoma cell line was harvested, washed, and resuspended in FACS buffer (PBS, 10% FBS, 0.1% NaN_3_) to a concentration of 5 × 10^6^ cells/mL. Cells The cells were incubated with primary antibodies against mGluR5 (1:250, Cat# ab27190, Abcam, Cambridge, UK) and CD30 (1:80, Ber-H2, Cat# M0751, Dako, Jena, Germany) for 2 h on ice. The cells were washed thrice with 1× PBS. The secondary antibodies (anti-rabbit-IgG antibody, Alexa Fluor 488, 1:1000, Cat# A-11008, Invitrogen, Waltham, MA, USA and anti-mouse antibody, Alexa Fluor 568, 1:250, Cat# A-11004, Invitrogen) were added and incubated for 2 h on ice. The labeled cells were washed thrice with 1× PBS, centrifuged each time at 500× *g* for 5 min at 4 °C, and resuspended in 500 μL of ice-cold FACS buffer for flow cytometry. The cells were double-labeled for Hodgkin lymphoma tumor marker CD30 and for cell surface receptor mGluR5. The gating strategy comprised the exclusion of dead cells and debris (forward and side scatter) as well as doublets (plotting height and width against area), and aimed for the double-positive (mGluR5^+^|CD30^+^) cells, using a fluorescence activated cell sorter (FACS) Aria II (Beckton Dickinson, Heidelberg, Germany).

### 2.8. Gene Expression Profiling

We extracted total RNA from all six classical Hodgkin lymphoma cell lines (5 × 10^5^ cells each) using the NucleoSpin^®^ NA Plus Kit (Macherey and Nagel, Düren, Germany). The quality of the RNA was controlled via the 2100 Bioanalyzer (Agilent Technologies, Santa Clara, CA, USA). RNA-sequencing was performed on a polyA^+^ enriched cDNA library on the BGISEQ-500 RNA-Sequencing pipeline (Beijing Genomics Institute, Hong Kong) yielding >40 Mio FASTQ 100 paired-end stranded reads that were quality controlled using FastQC v0.11.8 and then aligned to the human reference sequence (GRCh37.75) with the STAR 2.4.0.1 aligner [33]. The resulting BAM files were further investigated and normalized with the StringTie v2.2.1 pipeline [34,35], which yielded the relative mRNA quantities for each transcript present in the transcriptome dataset. As a quantitative measure for gene abundance, we used the FPKM value (fragments per kilobase of transcript per million mapped reads), which is calculated by the StringTie software. To control for multiple testing in the gene expression studies, we calculated the false discovery rate (FDR) [36]. Gene expression differences between the n = 1 “*GRM5* high-expressing” (L-1236) and the n = 5 “*GRM5* low-expressing” (L-428, L-540, SUP-HD1, KM-H2, HDLM-2) cell lines were considered significant if the up- or down-regulation was by a factor of five or larger and the FDR was <0.05. Differential gene expression yielded over 800 genes that were upregulated by >10-fold in L-1236. The genes of interest were annotated using the database for Annotation, Visualization, and Integrated Discovery (DAVID) tool [37].

### 2.9. Statistics

Quantitative data are presented as the mean ± SD of results obtained from at least three independent experiments. Significance levels of ICC signal variation, RT-qPCR relative expression levels, and Western blot densities were determined by one-way analyses of variance (ANOVA) and Tukey’s Multiple Comparison Test. A probability of < 0.05 was considered statistically significant, indicated in the graphs as *p* < 0.05 (*), *p* < 0.01 (**), *p* < 0.001 (***); n.s., not significant. Statistical analyses and graphing were performed with RStudio version 1.4.1106, and R version 4.2.2 software packages. Image J, and GraphPad Prism v9.2 (GraphPad Software, Inc., La Jolla, CA, USA).

## 3. Results

### 3.1. mGluR5 Is Expressed on Hodgkin Lymphoma Tissue at Varying Levels

To investigate a possible link between encephalitis and classical Hodgkin lymphoma, we performed immunostaining with anti-mGluR5 antibodies on tumor biopsy specimens from our patient with Ophelia syndrome and in n = 5 classical Hodgkin lymphoma patients without autoimmune encephalitis.

The index patient’s tumor revealed an intensive mGluR5 signal on classical Hodgkin lymphoma cells (Figure 1C). The controls showed a heterogeneous immunoreactivity pattern with far fewer positive cells and weaker intensities (Figure 1D–H). These initial findings prompted our further examination of mGluR5 expression in classical Hodgkin lymphoma cell lines.

### 3.2. Heterogeneous mGluR5 Expression Patterns

To characterize anti-mGluR5 staining intensities and distributions, as well as mGluR5/*GRM5* expression levels, we investigated six classical Hodgkin lymphoma cell lines. Jurkat cells served as controls. We performed immunofluorescence co-staining of mGluR5 and CD30 and were able to detect mGluR5 predominantly in the L-1236 and, to a lesser extent, in the KM-H2 cell lines. In the other cell lines, we mainly recorded low background fluorescence (Figure 2). Immunostaining of the L-1236 line revealed a specific distribution of the mGluR5 signals not seen in the other cell lines, with mGluR5 appearing to form a cluster on the cell surface, whereas a more even cell membrane distribution was seen in the KM-H2 line (100× magnification on Figure 2B; for 3D-reconstruction see Supplementary Figure S2). In the L-1236 and KM-H2 lines, anti-mGluR5 signals varied considerably, and cells that were anti-CD30 positive showed a tendency for higher anti-mGluR5 staining intensities (Figure 2A,B). The anti-CD30 staining also varied across the cell lines. The highest percentages of anti-CD30 positive cells were seen in the L-428 and L-540 lines, which had the lowest anti-mGluR5 signals (Figure 2).

In summary, immunostaining detected strong, moderate, and weak/absent staining intensities, depending on the cell line. Clearly detectable immune signals in the majority of cells were only seen in the L-1236 and KM-H2 cell lines. The overall staining intensities of the HDLM-2, SUP-HD1, L428, and L-540 lines were very low (in the range of background fluorescence). The distribution pattern of mGluR5 on the cell surface varied considerably between the different cell lines but also between single cells of the same line. A similar tendency of non-uniform staining, with the exception of the L-540 line, was seen for the CD30 marker. This staining pattern is typical for classical Hodgkin lymphoma and shows that not every tumor cell expresses this characteristic marker permanently [38,39].

**Figure 3:**
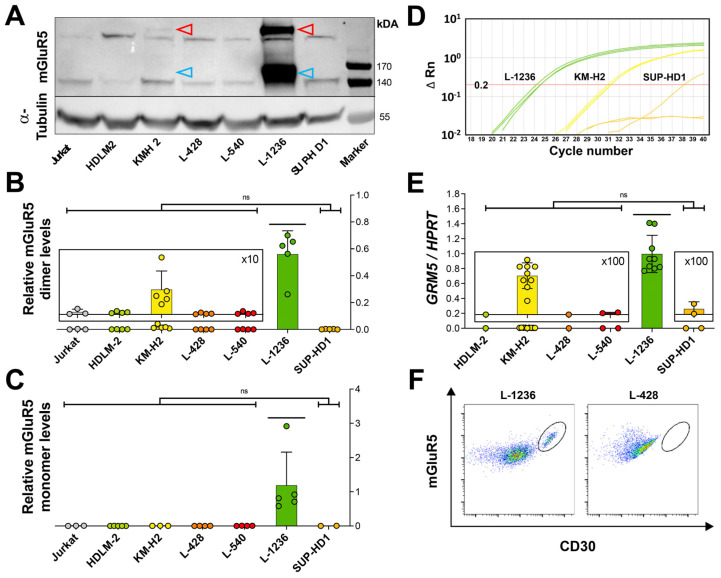
Relative mGluR5 protein levels and *GRM5* mRNA expression. (**A**) Representative Western blot with different mGluR5 expression levels on six classical Hodgkin lymphoma cell lines. The anti-mGluR5 antibody detects two specific bands: one band of higher molecular weight at ~280 kDa representing the mGluR5 dimer and its monomeric form at ~150 kDa. Signal intensities of dimeric (**B**) and monomeric (**C**) mGluR5 from three immunoblots were normalized to α-Tubulin expression. The L-1236 cell line (green) shows by far the highest expression of mGluR5. Jurkat cells serve as a negative control. (**D**) Amplification plot of RT-qPCR from classical Hodgkin lymphoma cell lines, depicting consistent amplification of the *GRM5* target gene only in the L-1236 and KM-H2 lines in all three RT-qPCR reactions and in SUP-HD1 in only one out of three RT-qPCR reactions with a high C*t* value. Fluorescence is plotted on a log-scale with ΔRn (horizontal lines) against cycle numbers (C*t* value) (vertical lines). The threshold for detection was set at ΔRn = 0.2. (**E**) Large variations in *GRM5* expression determined by RT-qPCR. L-1236 shows by far the highest amount of *GRM5* per *HPRT* mRNA copy number. (**F**) Representative dot plots from flow cytometry analysis with the six classical Hodgkin lymphoma cell lines. L-1236, but not L-428, shows a distinct mGluR5^+^|CD30^+^ subpopulation (gate) (after exclusion of dead cells and doublets). All bars represent the mean and SD of three independent experiments. ns = not significant. Significance values were calculated with one-way ANOVA followed by Tukey’s multiple comparison test.

To further quantify mGluR5/*GRM5* expression in classical Hodgkin lymphoma cells, we extended our investigations using immunoblotting and RT-qPCR (Figure 3A–E). On the protein level, we detected two major bands: one at ~150 kDa corresponding to the known monomeric form of mGluR5 and another at ~280 kDa corresponding to the disulfide-bond linked dimeric form of mGluR5 [40,41]. Considerably weaker bands at 140 kDa and 270 kDa were seen in all cell lines, including the Jurkat controls, and were thus regarded as non-specific (Figure 3A). Overall, mGluR5 protein abundance was by far highest in the L-1236 line (*p* < 0.0001) compared with all other classical Hodgkin lymphoma cell lines.

**Figure 4:**
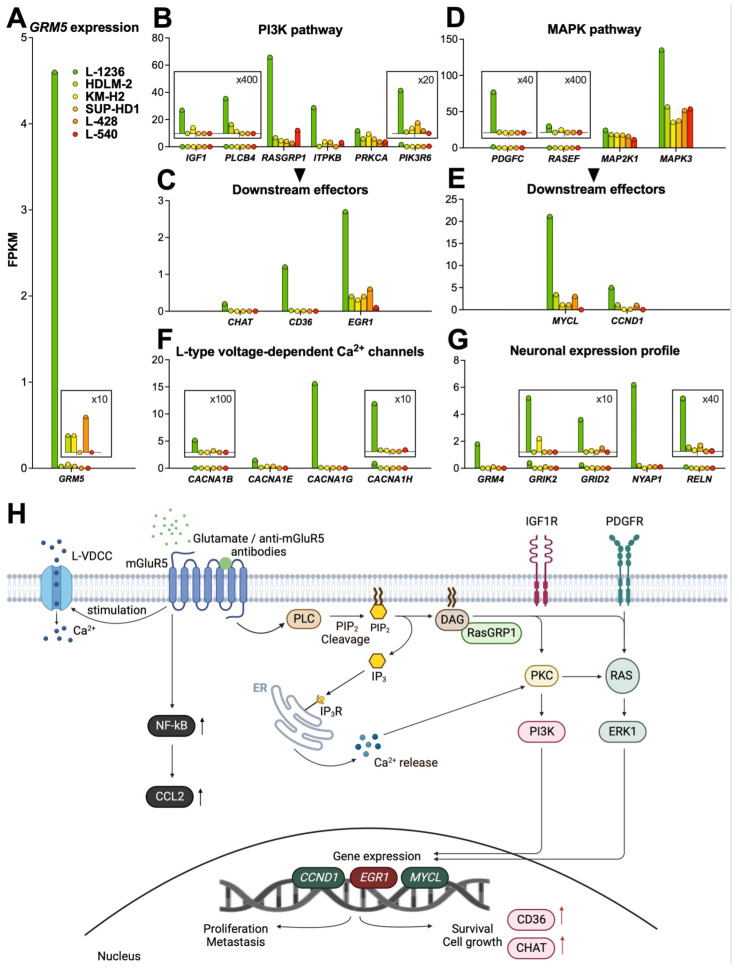
*GRM5*-related gene expression profile of classical Hodgkin lymphoma cell lines. RNA-sequencing data of classical Hodgkin lymphoma cell lines on *GRM5* expression and activation of mGluR5-related pathways. (**A**) L-1236 (green) shows by far the highest *GRM5* expression. Representative genes from PI3K (**B**) and MAPK (**D**) pathways with respective downstream effectors (**C**,**E**), calcium signaling (**F**), and neuronal expression profiles (**G**) are most activated in L-1236 (green). *Y*-axes are adjusted to considerably differing FPKM values. For details see magnified insets (scaling factors are indicated). (**H**) A schematic overview displays the known signal transduction pathways that are activated by mGluR5 in L-1236 according to our RNA-sequencing data. Expression of mGluR5 and activation by its corresponding ligands (e.g., L-glutamate or anti-mGluR5 antibodies) results in coupling with G-protein (not shown) and activation of protein lipase C (PLC). This is followed by the (over-)activation of signaling pathways promoting cell survival (PI3K), upregulating cell proliferation (MAPK), and increasing Ca^2+^-influx via L-type voltage-dependent Ca^2+^ channels (LVDCCs) leading to downstream modifications, all of which are implicated in classical Hodgkin lymphoma progression. CCND1, Cyclin D1; ChAT, Choline acetyltransferase; DAG, Diacylglycerol; EGR1, Early growth response 1; ERK1, Extracellular signal-regulated kinase 1; IP3, Inositol 1,4,5-triphosphate; IP3R, Inositol 1,4,5-triphosphate receptor; PIP2, Phosphatidylinositol 4,5-bisphosphate; PKC, Protein kinase C; PI3K, Phosphoinositide 3-kinase; RasGRP1, Ras guanyl-releasing protein 1. Created with BioRender.com.

In RT-qPCR, the mGluR5 encoding *GRM5* gene could be consistently amplified only in the two L-1236 and KM-H2 lines. In the RNA-sequencing experiment, this high variance in GRM5 mRNA copy numbers was confirmed (Figure 4A).

These findings encouraged us to proceed with flow cytometry to quantify mGluR5-positive cells in all six classical Hodgkin lymphoma cell lines in an unbiased manner. Looking at the FACS data from the L-1236 line, a subpopulation of double-positive (mGluR5^+^|CD30^+^) cells was easily distinguishable (Figure 3F), amounting to 10% of all viable cells. In the other classical Hodgkin lymphoma cell lines, either no distinct subpopulation of double-positive cells was found (L-428, HDLM-2), or the subpopulation was barely visible and the percentage of mGluR5^+^|CD30^+^ cells was significantly lower (below 6% for L-540, KM-H2, and SUP-HD1) (Figure 3F, Supplementary Figure S3).

Overall, immunohistochemistry, Western blot, RT-qPCR, and FACS consistently indicate heterogeneous mGluR5 expression, which was exceptionally high in the L-1236 line compared with the other classical Hodgkin lymphoma cell lines.

To verify the very heterogeneous *GRM5* expression in a larger number of classical Hodgkin lymphoma patients at the population level, we reanalyzed published gene expression data (Affymetrix microarray data) from a n = 130 cohort of patients with classical Hodgkin lymphoma [42]. In a subset of n = 6 patients (~5%), we detected a high expression of *GRM5* (Supplementary Figures S2 and S4). These results confirm the findings from our analysis of classical Hodgkin’s lymphoma cell lines, but do not allow us to draw conclusions about the exact pathophysiology of Ophelia syndrome because we lack the anamnestic and clinical data from this large cohort, particularly with regard to the presence of autoimmune encephalitis.

### 3.3. mGluR5 Expression Is Correlated with Sustained Activation of Glutamatergic Signaling Pathways

To investigate whether the highly variant mGluR5 expression would have an effect on cell physiology, we performed RNA-sequencing of all six classical Hodgkin lymphoma cell lines. The gene expression profile of L-1236 was markedly different from that of the other cell lines, and data analysis revealed that together with *GRM5*, more than 800 genes were upregulated (≥10-fold). However, comparing the expression pattern of a single cell line (L-1236 in this case) with five other cell lines could lead to random results that would not have occurred if more cell lines of each type had been examined. Therefore, the results below should be interpreted with caution.

For further analysis, we grouped the genes by G-protein coupled receptor (GPCR)-related signaling pathways (Supplementary Table S3). Most strikingly, several genes involved in mGluR5-linked signal transduction were overexpressed, indicating hyperactivity of two fundamental pathways (PI3K and MAPK) that control many processes essential for tumor growth and survival (Figure 4A–H). In the following paragraphs, we depict the fold increase in mRNA transcript numbers (given as FPKM values) of L-1236 over the average expression in the remaining n = 5 classical Hodgkin lymphoma cell lines. In summary, we identified upregulation of several genes, particularly of the PI3K and MAPK pathways, as a characteristic signature of the L-1236 cell line.

#### 3.3.1. PI3K Pathway

We detected upregulation of *PLCB4* (Phospholipase Cβ4, 15.6-fold upregulation), which is directly involved in mGluR5-related signal transduction by cleaving phosphatidylinositol-4,5-diphosphate (PIP2) into inositol 1,4,5-triphosphate (IP3) and diacylglycerol (DAG) [43,44]. The DAG-regulated nucleotide exchange factor RASGRP1 (Ras guanyl releasing protein 1) specifically activates Ras [45]. IP3 binds to its corresponding receptor (IP3R) at the endoplasmic reticulum (ER) resulting in Ca^2+^ release into the cytoplasm [43]. Both the elevated intracellular Ca^2+^ and DAG activate protein kinase C (PKC), which in turn activates Ras, followed by ERK1 phosphorylation. We found upregulation of *RASGRP1* (11.1-fold upregulation), *ITPKB* (Inositol 1,4,5-triphosphate receptor, IP3R; 13.9-fold upregulation) and *PIK3R6* (Phosphoinositide 3-kinase, PI3K; 10.1-fold upregulation). Elevated gene expression of the downstream targets *EGR1* (Early growth response 1, EGR1; 7.5-fold upregulation), *CHAT* (Choline acetyltransferase, ChAT; 112.4-fold upregulation) and *CD36* (208.5-fold upregulation) confirmed activation of the PI3K pathway.

Growth factors are key constituents that help sustain G-protein coupled receptor (GPCR) signaling. *IGF1* (Insulin-like growth factor, IGF1; 15.4-fold upregulation) is an upstream regulator of PI3K and was upregulated as well, suggesting receptor binding and activation upon enhanced mGluR5 expression (Figure 4B,C).

#### 3.3.2. MAPK Pathway

The MAPK cascade is overactive in about one-third of all human cancers [46]. Inhibition of components of this cascade by targeted inhibitors represents an important antitumor strategy [46]. mGluR5-dependent overexpression was found in *PDGFC* (platelet-derived growth factor C, 332.7-fold upregulation), *RASEF* (RAS and EF-hand domain containing protein, 13.4-fold upregulation), as well as downstream effectors *MAP2K1* (mitogen-activated protein kinase kinase 1, MEK1, 1.5-fold upregulation), *MAPK3* (extracellular signal-regulated kinase 1, ERK1, 2.9-fold upregulation), nuclear transcription targets *MYCL* (L-Myc proto-oncogene BHLH transcription factor, 12.4-fold upregulation), and *CCND1* (cyclin D1, 10.7-fold upregulation) that are all involved in the MAPK pathway (Figure 4D,E).

#### 3.3.3. Calcium Signaling

Activation of mGluR5 followed by membrane depolarization through Ca^2+^-release opens L-type voltage-dependent Ca^2+^-channels (L-VDCCs) [47]. This implies a crosstalk between mGluRs, intracellular Ca^2+^ and membrane Ca^2+^-channels.

We found L-VDCC genes, including *CACNA1B* (29.7-fold upregulation), *CACNA1E* (10.5-fold upregulation), *CACNA1G* (137.5-fold upregulation), and *CACNA1H* (54.4-fold upregulation) (Calcium voltage-gated channel subunit α-1B, E, G, and H, respectively) were upregulated in L-1236 along with high *GRM5* expression (Figure 4F).

#### 3.3.4. NF-kB Pathway

Classical Hodgkin lymphoma is also characterized by a high constitutive activity of the NF-kB pathway. Examining genes involved in this pathway, we found a significant overexpression of *CCL2* (C-C chemokine ligand 2, 14.4-fold upregulation, Supplementary Table S3) in L-1236.

## 4. Neuronal Expression Profile

Associated with a high expression of *GRM5*, we detected upregulation of other genes involved in glutamatergic signaling, including *GRM4*, which is a member of group III mGluRs. In contrast to the other cell lines, L-1236 expressed ionotropic glutamate receptors (iGluRs), including *GRIK2* (glutamate ionotropic receptor kainate type subunit 2) and *GRID2* (glutamate ionotropic receptor delta type subunit 2). Interestingly, genes normally exclusively turned on in neurons, were also upregulated in L-1236. These included *NYAP1* (neuronal tyrosine-phosphorylated phosphoinositide 3-kinase adaptor 1) that regulates neuronal morphogenesis and *RELN* (Reelin, extracellular matrix glycoprotein), a regulator of neuronal migration, which also activates N-methyl-D-aspartate receptors (NMDARs) and α-amino-3-hydroxy-5-methyl-4-isoxazole propionic acid receptors (AMPARs) (Figure 4G) [48,49].

## 5. Discussion

Previous work has focused on the identification of mGluR5 as a neuronal antigen target of autoantibodies in Ophelia syndrome, a disorder characterized by classical Hodgkin lymphoma in association with anti-mGluR5 mediated encephalitis [18,19]. However, little attention has been paid to the question, whether mGluR5 can be found in classical Hodgkin lymphoma tumor tissue. Thus, the aim of this study was to search for mGluR5 expression in classical Hodgkin lymphoma cells and gain a more in-depth understanding of the link between tumor growth and autoimmunity.

In neurons, stimulation of mGluR5 activates the PI3K and MAPK pathways and causes increased Ca^2+^-influx into the cytosol [50,51]. In classical Hodgkin lymphoma, these glutamatergic signaling cascades are the most frequently dysregulated ones [52,53,54], with glutamate stimulating proliferation and migration of tumor cells via GPCR activation [44].

Our study shows that the two fundamental pathways PI3K and MAPK with PDGFC (platelet-derived growth factor C), in conjunction with the increased recruitment of its receptor PDGFR (platelet-derived growth factor receptor), which in turn also enhances MAPK signaling [55,56], were upregulated along with high mGluR5 expression in classical Hodgkin lymphoma cell lines. In addition, we detected upregulation of downstream targets of the PI3K and MAPK pathways that are known to be generally involved in tumor progression (Figure 4H). High expression of the surface protein CD36 [57], the transcription factors Early Growth Response Protein 1 [58], Cyclin D1 [59], and L-Myc [60,61] and of the enzyme ChAT [62,63] have been linked to tumor progression, poor survival and increased metastasis in several cancers, including melanoma [59] and recently also in lymphoma [60,61].

ChAT is a critical enzyme for acetylcholine synthesis. Acetylcholine can be produced in lung and colon cancer cells and acts as an autocrine and paracrine growth factor [62,63]. High *CHAT* overexpression (≥100-fold), as shown in L-1236, may result in non-neuronal production and release of acetylcholine by Hodgkin and Reed–Sternberg cells, thereby self-promoting lymphoma growth.

Transcription factor L-Myc, a member of the Myc-proto-oncogene family, is amplified and overexpressed in 70% of all human malignancies including classical Hodgkin lymphoma. Its upregulation contributes to uncontrolled cell proliferation, survival, and escape from immune surveillance [60,61]. As classical Hodgkin lymphoma shares many characteristics with acute inflammatory processes, NF-kB signaling plays a major role by mediating the immediate-early expression of various cytokines including the chemokine CCL2 [56]. Upregulation of *CCL2*, as shown in our data (Supplementary Table S3), has been associated with cancer advancement, metastasis, and relapse [64,65].

In addition to this, other glutamate receptors are differentially expressed in tumors [66,67,68], e.g., mGluR1 in melanoma [55] or mGluR3 in malignant gliomas and mGluR4 in medulloblastomas [66]. Activation of these receptors stimulates tumor growth and development of metastasis in a MAPK-dependent manner [66]. Moreover, investigations showed that glutamate receptor antagonists, including the potent mGluR3 antagonist LY341495, limit tumor growth [66,69]. Although mGluR5 was not considered an oncogene previously, it has recently been found to play an important role in promoting tumor growth, e.g., in melanoma and oral squamous cell carcinoma [70,71,72], and to have an influence on astrocyte proliferation upon chemical and mechanical injury [73].

Besides these data on various tumors, non-malignant immune cells, e.g., B- and T-lymphocytes also show baseline expression of mGluR5 supporting our findings [74]. This indicates that the basic machinery required for mGluR5 signaling is already present prior to the development of Hodgkin and Reed–Sternberg cells and the transformation to classical Hodgkin lymphoma. Interestingly, our analysis also showed several neuronal genes activated in L-1236, suggesting that some classical Hodgkin lymphoma cells might exploit neuronal and neurodevelopmental pathways for tumorigenesis. Consistent with this, studies have reported that tumors behave more aggressively when expressing genes related to the nervous system [75,76].

The six classical Hodgkin lymphoma cell lines L-1236, HDLM-2, KM-H2, SUP-HD1, L-428, and L-540 investigated in this study were established between the 1970s and 1990s. They have been used extensively as model systems in research for many years and are considered as “the classical Hodgkin lymphoma cell lines” [77]. The L-1236 line was obtained from the peripheral blood of a 31-year-old patient diagnosed with classical Hodgkin lymphoma of mixed cellularity subtype, who developed an advanced disease with rapid progression and multiple relapses [28]. Unlike our index patient with Ophelia syndrome, no neurologic or psychiatric symptoms suggestive of encephalitis were reported from the L-1236 donor [28]. However, data on the clinical course and medical history of that patient are incomplete and signs of previous paraneoplastic neurological symptoms may have gone unnoticed or unreported. In any case, both our patient and the L-1236 donor suffered from advanced stage disease, an aggressive clinical course. The strong expression of mGluR5 and its downstream effectors (e.g., *MYCL* and *EGR1*) may have contributed to the aggressive clinical course.

Given the observed occurrence of classical Hodgkin lymphoma in the wake of half of all reported Ophelia syndromes and reports of additional cases of classical Hodgkin lymphoma with preceding paraneoplastic symptoms before [78,79,80,81], we believe it is possible that mGluR5 expression on classical Hodgkin lymphoma cells not only drives tumor progression but also plays a role in triggering anti-mGluR5 encephalitis early during tumor development, long before classical Hodgkin lymphoma is clinically detected.

However, classical Hodgkin lymphoma does not follow in all cases of anti-mGluR5 encephalitis, and conversely, Ophelia syndrome occurs in only a minority of patients with classical Hodgkin lymphoma. According to our reanalysis of a large mRNA expression dataset of Hodgkin tumor tissues, high mGluR5 expression does not seem to be all that rare in classical Hodgkin lymphoma. We hypothesize that other factors must play a role in the manifestation of Ophelia syndrome. Such factors may include the histological subtype, mechanisms of immune tolerance or preceding viral infections, which may trigger both encephalitis and subsequent tumor progression as a “second hit”. Further prospective studies are needed in cases of paraneoplastic neurological disease and/or classical Hodgkin lymphoma with respect to mGluR5 expression on tumor cells.

## 6. Conclusions

In recent years, significant progress has been made in our understanding of Ophelia syndrome. However, the underlying molecular mechanisms leading to both anti-mGluR5 encephalitis and classical Hodgkin lymphoma were poorly understood. This study contributes to their understanding and may have clinical implications.

Detection of paraneoplastic neurological disease preceding classical Hodgkin’s lymphoma can be challenging [21]. However, early diagnosis of an Ophelia syndrome is paramount both for the treatment of anti-mGluR5 encephalitis and the earliest possible recognition of a subsequent classical Hodgkin lymphoma. Based on our findings, we recommend (i) thorough follow-up and close screening for classical Hodgkin lymphoma after detection of anti-mGluR5 encephalitis, (ii) searching for mGluR5 expression in classical Hodgkin lymphoma biopsy material of patients with autoimmune encephalitis, and (iii) inclusion of mGluR5 screening alongside the standard diagnostic procedure for classical Hodgkin lymphoma as this could provide further information on tumor progression and may serve as a prognostic marker.

Other factors may also be implicated in the pathogenesis of the Ophelia syndrome in the setting of classic Hodgkin lymphoma, because mGluR5 expression is not so rare in classic Hodgkin lymphoma, in contrast to the rarity of the Ophelia syndrome. Therefore, further studies on the clinical relevance of mGluR5 expression in classical Hodgkin lymphoma, as well as its functional impact on tumor development are needed to gain a more in-depth understanding of the pathogenesis of anti-mGluR5 encephalitis and its link to lymphoma development.

## Data Availability

The raw FASTQ sequence files of all the RNA-sequencing runs as well as the StringTie result files have been deposited in NCBI’s Gene Expression Omnibus [82] and are accessible through GEO Series accession number GSE212326 “https://www.ncbi.nlm.nih.gov/geo/query/acc.cgi?acc=GSE212326, accessed on 12 February 2023”.

## Supplementary Materials

The following supporting information can be downloaded at: https://www.mdpi.com/article/10.3390/cells12040606/s1. Figure S1: mGluR5 banding pattern of human Hodgkin cells and mouse brain. Figure S2: Distribution of mGluR5 on the surface of Hodgkin lymphoma cells—3D-reconstruction. Figure S3: FACS analysis of classical Hodgkin lymphoma cells. Figure S4: Calculation of *GRM5* gene expression intensities using the public GEO GSE17920 dataset. Figure S5: mRNA expression levels of housekeeping genes in all six classic Hodgkin lymphoma cell lines. Table S1: Primers used in RT-qPCR. Table S2: Clinical details of classical Hodgkin lymphoma patients from GEO GSE17920 with high *GRM5* expression. Table S3: Main upregulated signaling pathways in the L-1236 cell line. Information about the source and character of the investigated classic Hodgkin lymphoma cell lines here.
