# Supplementary material for: Hodgkin Lymphoma Cell Lines and Tissues Express mGluR5: A Potential Link to Ophelia Syndrome and Paraneoplastic Neurological Disease
Source: Cells. 2023 Feb 13;12(4):606. doi: 10.3390/cells12040606 (PMC9953995; doi:10.3390/cells12040606)
Supplement: Supplementary file 1 [file cells-12-00606-s001.zip › cells-2146769-supplementary.pdf]

Supplemental Material

Hodgkin lymphoma cell lines and tissues express mGluR5: a potential link to Ophelia syndrome and paraneoplastic neurological disease

Sofia Schnell, Ellen Knierim, Petra Bittigau, Jakob Kreye, Kathrin Hauptmann, Patrick Hundsdoerfer, Susanne Morales-Gonzalez, Markus Schuelke, Marc Nikolaus

Figure S1

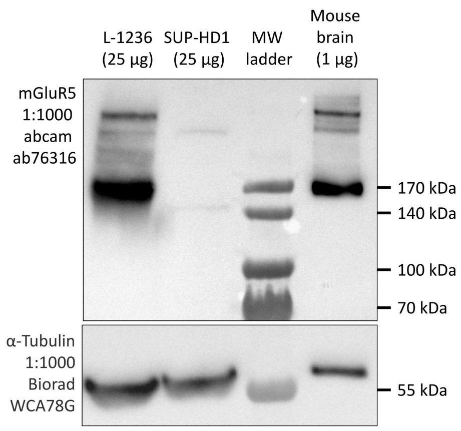

Figure S1: mGluR5 banding pattern of human Hodgkin cells and mouse brain. Representative Western blot with 25 µg protein homogenates from the L-1236 and SUP-HD1 classical Hodgkin lymphoma cell lines and with 1 µg from mouse brain (positive control) side-by-side that shows a **characteristic mGluR5 banding pattern with two specific bands: one band of higher molecular weight at ~280 kDa representing the mGluR5 dimer and its monomeric form at ~150 kDa.** The mGluR5 expression in mouse brain is much higher than in the L-1236 cell line, *nota bene* the different loading quantities between mouse brain and Hodgkin cells (see α-Tubulin banding intensities).

Figure S2

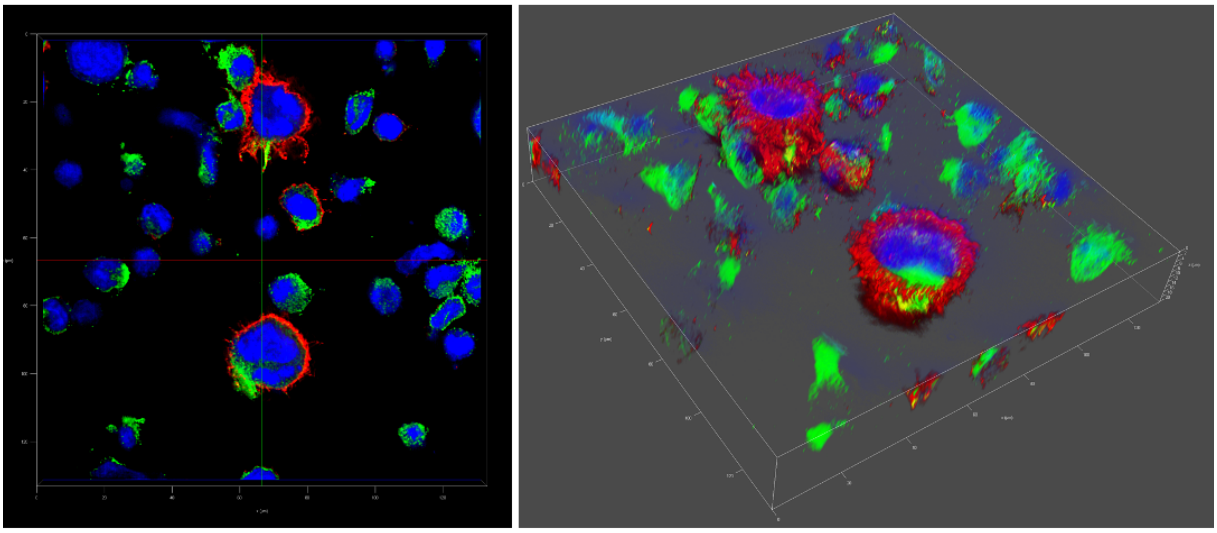

Figure S2: Distribution of mGluR5 on the surface of Hodgkin lymphoma cells - 3D-reconstruction. Immunofluorescence co-staining of mGluR5 (green) and CD30 (red) on classic Hodgkin lymphoma cell line L-1236. The anti-mGluR5 signal was most intense in comparison to all other cell lines and revealed a specific distribution of mGluR5 appearing to form clusters in individual membrane regions of each cell surface. 3D reconstruction from a 100x magnification stack of 96 images.

Figure S3

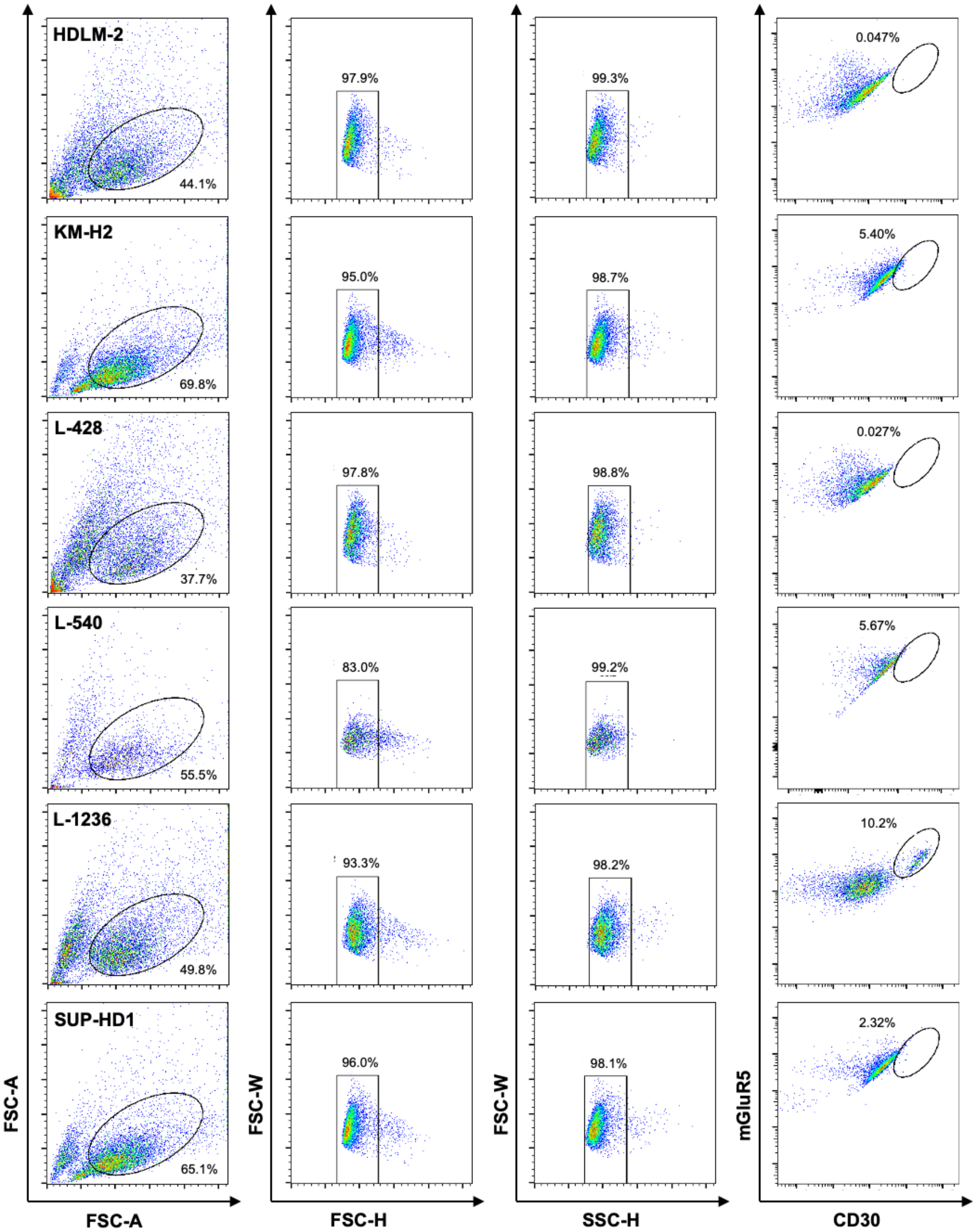

Figure S3: FACS analysis of classical Hodgkin lymphoma cells. Representative dot plots from flow cytometry with FACS ARIA II aiming for double-positive $CD30^{+}|mGluR5^{+}$ Hodgkin lymphoma cells. Gating strategy comprised separation of dead cells and debris by size (forward scatter, FSC) and granularity (side scatter, SSC), following the exclusion of doublets by height and width on FSC and SSC with the height as signal duration (FSC-H) and the width as speaksignal (FSC-W). Gating for $CD30^{+}|mGluR5^{+}$ cells (depicted on log scale) revealed a clearly distinct subpopulation of double-positive Hodgkin lymphoma cells in L-1236, which was barely delineable in KM-H2, L-540 and SUP-HD1, and not detectable in L-428 and HDLM-2.

Figure S4

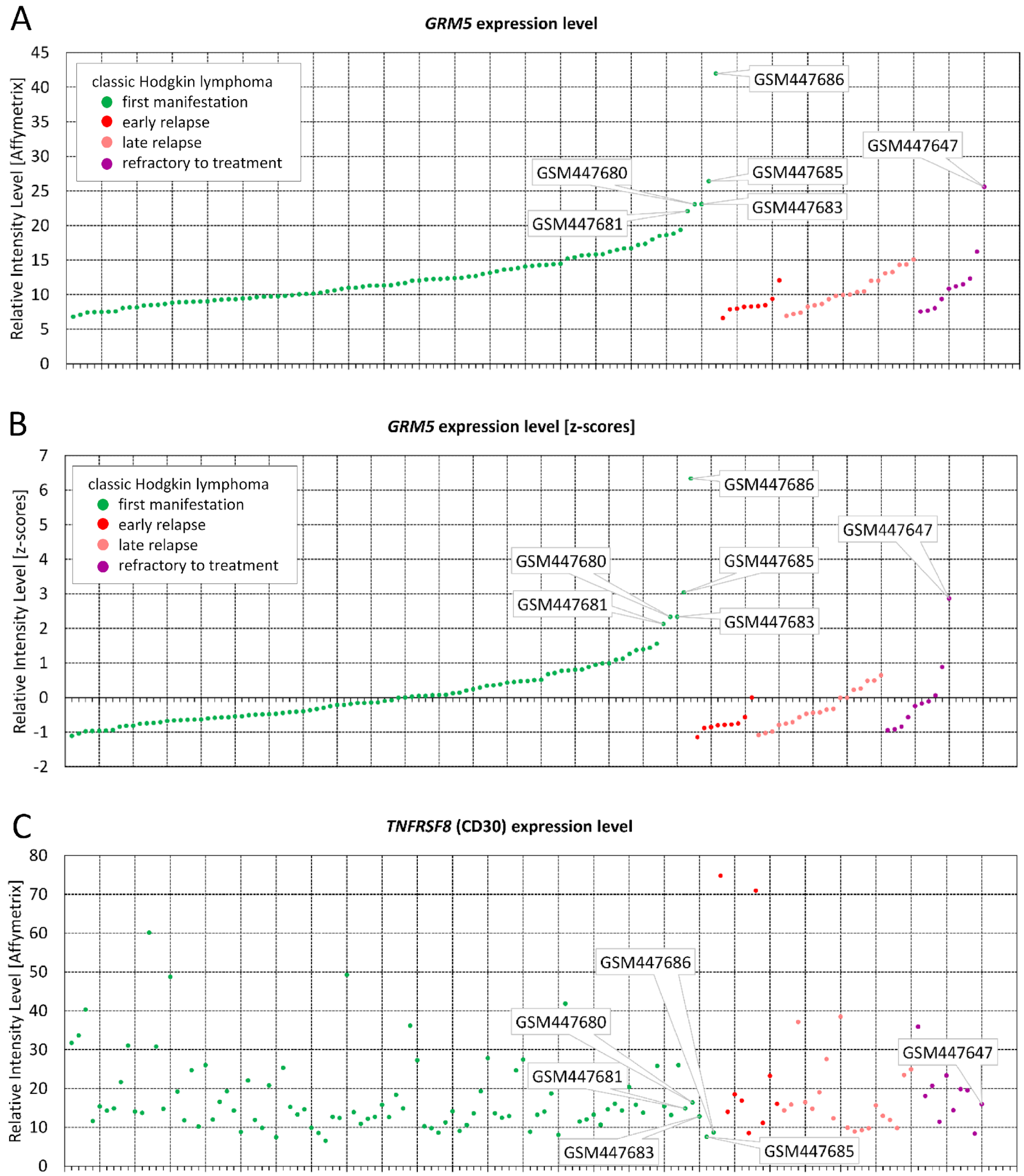

Figure S4: Calculation of *GRM5* gene expression intensities using the public GEO GSE17920 dataset. Relative *GRM5* and *CD30* gene expression intensities were recalculated from the raw data of a n=130 cohort of patients with classical Hodgkin lymphoma [1], sorted by increasing *GRM5* expression levels, and grouped according to "primary manifestation" *versus* "early" and "late relapse" and "refractory to treatment". The clinical details of the indicted individuals are reported in **Table S2.**

Table S1

Transcript-specific oligonucleotide primers for RT-qPCR					
**Gene**	**Forward primer**	**Reverse primer**	**PCR cycles**	**Annealing Tm [°C]**	**Length [bp]**
GRM5	5-TGTGCAGTGAACCATGTGAG-3	5-GGGATCAAGTCACAACCTGT-3	40	60	178
HPRT	5-ACAATGCAGACTTTGCTTTCC-3	5-TCAAGGGCATATCCTACAACAA-3	40	60	140

Table S1: Primers used in RT-qPCR

Table S2

Clinical detail of classical Hodgkin lymphoma patients							
**GEO accession**	**Tissue type**	**Disease status**	**Manifestation**	**Age [years]**	**Sex**	**Disease stage**	**IPS**
GSM447681	lymphoma	chronic Hodgkin lymphoma	first manifestation	38	F	2	1
GSM447680	lymphoma	chronic Hodgkin lymphoma	first manifestation	22	F	2	2
GSM447686	lymphoma	chronic Hodgkin lymphoma	first manifestation	27	F	2	2
GSM447685	lymphoma	chronic Hodgkin lymphoma	first manifestation	47	M	2	3.5
GSM447683	lymphoma	chronic Hodgkin lymphoma	first manifestation	24	M	2	2
GSM447647	lymphoma	chronic Hodgkin lymphoma	refractory to treatment	78	M	3	4.7

Table S2: Clinical details of classical Hodgkin lymphoma patients from the GEO GSE17920 study with high *GRM5* expression in their tumor cells, IPS, international prognostic score.

Table S3

Main upregulated signaling pathways in the L-1236 cell line		
**PI3K/AKT pathway**	**Gene**	**Upregulation** [fold change]
• Metabotropic glutamate receptor 5 (mGluR5)	*GRM5*	>270-fold
• Phospholipase C beta 4 (PLCβ4)	*PLCB4*	>15-fold
• Ras guanyl releasing protein 1 (Ras GRP1)	*RASGRP1*	>11-fold
• Inositol 1,4,5-triphosphate receptor (IP_3R)	*ITPKB*	>13-fold
• Protein kinase C (PKC)	*PRKCA*	>2-fold
• Insulin growth factor 1 receptor (IGF1R)	*IGF1R*	>15-fold
• Phosphatidylinositol 3-kinase (PI3K)	*PIK3R6*	>10-fold
• Early growth response 1 (Egr1)	*EGR1*	>8-fold
• CD36	*CD36*	>208-fold
• ChAT	*CHAT*	>112-fold
**MEK/ERK pathway**		
• Platelet-derived growth factor C (PDGFC)	*PDGFC*	>332-fold
• Ras and EF-hand domain containing protein	*RASEF*	>13-fold
• Extracellular signal-regulated kinase 1 (ERK1)	*MAPK3*	> 3-fold
• MycL	*MYCL*	>12-fold
• Cyclin D1	*CCND1*	>10-fold

**L-type voltage-dependent Ca2+ channels (LVDCCs)**		
• Ca^{2+} voltage-gated channel subunit alpha1 B (CACNA1B)	*CACNA1B*	>30-fold
• Ca^{2+} voltage-gated channel subunit alpha1 E (CACNA1E)	*CACNA1E*	>11-fold
• Ca^{2+} voltage-gated channel subunit alpha1 G (CACNA1G)	*CACNA1G*	>273-fold
• Ca^{2+} voltage-gated channel subunit alpha1 H (CACNA1H)	*CACNA1H*	>54-fold
**NF-kB signaling**		
• C-C chemokine ligand 2	*CCL2*	>361-fold
**Neuronal expression profile**		
• Metabotropic glutamate receptor 4 (mGluR4)	*GRM4*	>71-fold
• Glutamate ionotropic receptor kainate type subunit 2 (GluK2)	*GRIK2*	>23-fold
• Glutamate ionotropic receptor delta type subunit 2 (Glu-R delta 2)	*GRID2*	>25-fold
• Neuronal tyrosine-phosphorylated phosphoinositide 3-kinase adaptor 1	*NYAP1*	>72-fold
• Reelin	*RELN*	>19-fold

Table S3: Main upregulated signaling pathways in the L-1236 cell line.

Figure S5

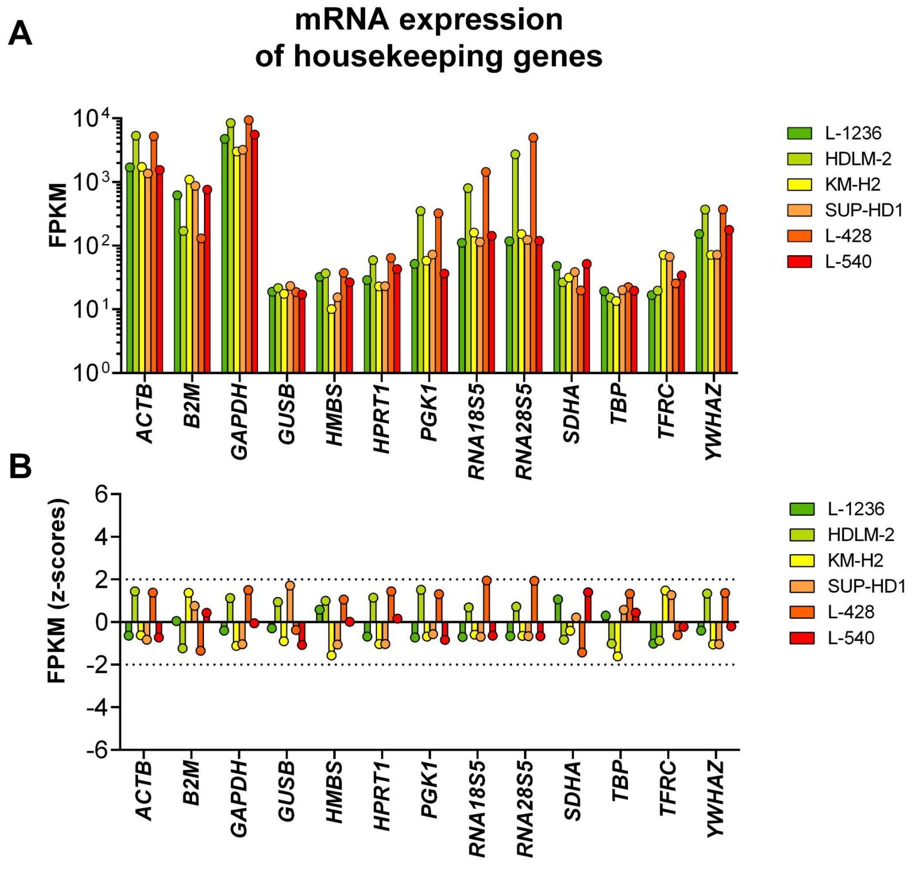

Figure S5: (**A**) In order to compare the basic housekeeping gene mRNA expression levels, we plotted the absolute FPKM values of all six CHL cell lines side-by-side. (**B**) Z-transformation of the FPKM-values for the single cHL cell lines confirms that the housekeeping gene mRNA expression levels of all six cHL cell lines are located within ±2 standard deviations around the mean. This excludes uneven gene expression as confounder for the differences in gene expression as shown on **Figure 4**. ***FPKM***, Fragments Per Kilobase Million, ***ACTB***, actin beta; ***B2M***, beta-2-microglobulin; ***GAPDH***, glyceraldehyde-3-phosphate dehydrogenase; ***GUSB***, glucuronidase beta; ***HMBS***, hydroxymethylbilane synthase; ***HPRT1***, hypoxanthine phosphoribosyltransferase 1; ***PGK1***, phosphoglycerate kinase 1; ***RNA18S5***, 18S rRNA; ***RNA28S5***, 28S rRNA; ***SDHA***, succinate dehydrogenase complex subunit A; ***TBP***, TATA box binding protein; ***TFRC***, transferrin receptor; ***YWHAZ***, Tyrosine 3-monooxygenase/tryptophan 5-monooxygenase activation protein zeta polypeptide

Source of the cHL cells

The cHL cells have been obtained from a controlled cell repository (Leibniz-Institute DSMZ–German Collection of Microorganisms and Cell Cultures) and have been cultured according to their protocols. Below we provide the links to this repository, so that the readers can obtain more information on these cell lines with regard to cell donors, cytogenetics, immunology markers, morphology, and culture conditions.

**L-1236**	https://www.dsmz.de/collection/catalogue/details/culture/ACC-530
**L-428**	https://www.dsmz.de/collection/catalogue/details/culture/ACC-197
**HDLM-2**	https://www.dsmz.de/collection/catalogue/details/culture/ACC-17
**KM-H2**	https://www.dsmz.de/collection/catalogue/details/culture/ACC-8
**L-540**	https://www.dsmz.de/collection/catalogue/details/culture/ACC-72
**SUP-HD1**	https://www.dsmz.de/collection/catalogue/details/culture/ACC-574

Literature

[1] C. Steidl, T. Lee, S.P. Shah, P. Farinha, G. Han, T. Nayar, A. Delaney, S.J. Jones, J. Iqbal, D.D. Weisenburger, M.A. Bast, A. Rosenwald, H.-K. Muller-Hermelink, L.M. Rimsza, E. Campo, J. Delabie, R.M. Braziel, J.R. Cook, R.R. Tubbs, E.S. Jaffe, G. Lenz, J.M. Connors, L.M. Staudt, W.C. Chan, R.D. Gascoyne, Tumor-associated macrophages and survival in classic Hodgkin's lymphoma, N. Engl. J. Med. 362 (2010) 875–885. https://doi.org/10.1056/NEJMoa0905680.
